# Disc degeneration implies low back pain

**DOI:** 10.1186/s12976-015-0020-3

**Published:** 2015-11-09

**Authors:** Chang-Jiang Zheng, James Chen

**Affiliations:** School of Public Health, 36 Nathan Lane North, Minneapolis, MN 55441 USA; College of Science and Engineering, University of Minnesota, Minneapolis, MN 55455 USA

**Keywords:** Disc degeneration, Low back pain, Causality, Dynamic modeling, Age

## Abstract

**Background:**

Low back pain exerts a tremendous burden on individual patients and society due to its prevalence and ability to cause long-term disability. Contemporary treatment and prevention efforts are stymied by the absence of a confirmed cause for the majority of low back pain patients.

**Methods:**

A system dynamics approach is used to build a physiologically-based model investigating the relationship between disc degeneration and low back pain. The model’s predictions are evaluated under two different types of study designs and compared with established observations on low back pain.

**Results:**

A three-compartment model (no disc degeneration, disc degeneration with pain remission, disc degeneration with pain recurrence) accurately predicts the age-specific prevalence observed in one of the largest population-based surveys (*R*^2^ = 0.998). The estimated transition age at which intervertebral discs lose the growth potential and begin degenerating is 13.3 years. The estimated disc degeneration rate is 0.0344/year. Without any additional change being made to parameter’s values, the model also fully accounts for the age-specific prevalence of disc degeneration detected with a lumbar MRI among asymptomatic individuals (*R*^2^ = 0.978).

**Conclusions:**

Dual testing of the proposed mechanistic model with two independent data sources (one with lumbar MRI and the other without) confirm that disc degeneration is the driving force behind and cause of age dependence in low back pain. Observed complexity of low back pain epidemiology arises from the slow dynamics of disc degeneration coupled with the fast dynamics of disease recurrence.

## Background

Lumbar intervertebral disc degeneration is nearly ubiquitous among patients with symptomatic back pain. However, a causal relationship between disc degeneration and low back pain has yet to be established (Lutz et al. 2003). When asymptomatic individuals are examined using lumbar MRI, disc degeneration is commonly found (Boden et al. 1990; Jensen et al. 1994; Powell et al. 1986). Conventional thinking suggests that, if disc degeneration played a significant role in the etiology of low back pain, disc degeneration should be uncommon in asymptomatic individuals.

In this communication, we provide a mechanistic explanation for the asymptomatic-disc-degeneration conundrum and propose that disc degeneration is the predominant cause of low back pain. The key to solving the apparent conundrum is to understand that most case–control studies rely on two fundamental assumptions. The first assumption is that the disease mechanism is non-recurrent, or in other words, study subjects are assumed to start in a disease-free state and progress through certain irreversible biological mechanisms into a terminal state. When a case of clinical interest is ascertained, she (or he) is identified through the occurrence of a terminal event (also called an incident event). Secondly and more importantly, all accepted controls must still remain at risk at the time of sampling (i.e., they have continuously been event-free from the beginning) (Rodrigues and Kirkwood 1990).

The conventional causality inference framework imposed by a traditional case–control study is impractical or inappropriate for studying the etiology of low back pain. The natural history of low back pain shows not only age dependence, but also a pattern of remission and recurrence which is different from other irreversible clinical outcomes such as cancer, stroke or heart attack. When a patient experiences a low back-pain episode, she (or he) will eventually recover from it. As the patient ages, she (or he) will experience additional episodes and not remember when the first episode occurred. Because the onset time of the first-ever pain episode is rarely known, any newly reported pain symptoms during a specified study interval cannot be reliably viewed as an incident event. Even more ominously, the absence of a pain episode during the same time interval cannot be accepted as evidence that the subject has never had a back pain episode in the past. So to truly understand the root cause of low back pain, the most feasible and most appropriate approach is to quantify and correct for the recurrent rhythm of low back pain rather than to rely on our constrained ability to ascertain incident events and even more elusive controls.

In the following communication, we use a system-dynamics method to investigate how disc degeneration relates to low back pain and to generate testable predictions. We begin our study with the construction of a physiologically-based three-compartment model to conceptualize the age-dependent population dynamics of disc degeneration and low back pain. After that, we derive two highly verifiable predictions. The first one relates to the population-based age-dependent prevalence of low back pain; the second one describes the age-specific percentage of disc degeneration among asymptomatic individuals. Testing these dual predictions with published studies allows us to confirm the existence of a causal relationship between disc degeneration and low back pain.

## Methods

### Constructing the model

Three clinically distinguishable compartments are assumed to exist:

1. *N*: Individuals who reside in compartment *N* have normal intervertebral discs and experience no back pain symptoms. At age *t*, the compartment’s size (the number of individuals remaining in compartment *N*) is *x* (*t*).
2. *D*_0_: Individuals who reside in compartment *D*_0_ have disc degeneration although they do not currently experience any back pain symptoms. At age *t*, the compartment’s size (the number of individuals remaining in compartment *D*_0_) is *y* (*t*).
3. *D*_1_: Individuals who reside in compartment *D*_1_ not only have disc degeneration but also have ongoing back pain. At age *t*, the compartment’s size (the number of individuals remaining in compartment *D*_1_) is *z* (*t*).
  Kinetic relationships among the three compartments are schematically diagramed in Fig. 1. The transitions between them are defined as follows:

**Fig. 1:**
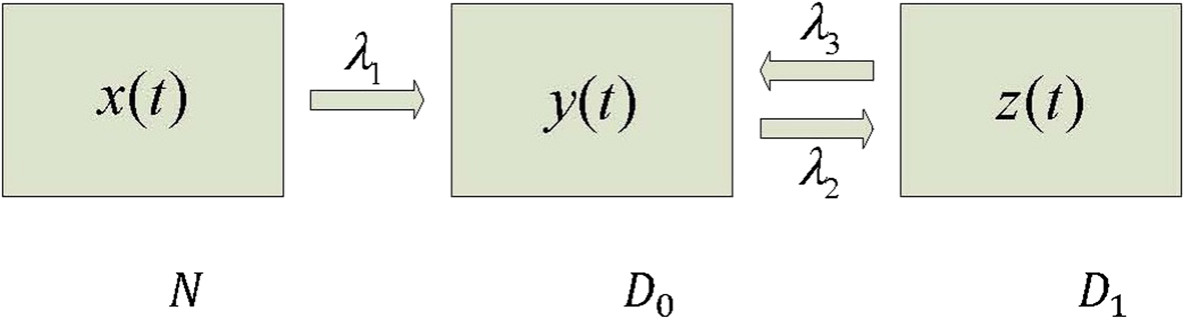
A three-compartment flow chart for the dynamic back-pain model. Individuals without disc degeneration initially reside in compartment *N*. Once having disc degeneration, they are moved to compartment *D*_0_. This is then followed by frequent interchange between compartments *D*_0_ and *D*_1_
4. *N* → *D*_0_: Normal individuals can move from compartment *N* to compartment *D*_0_ with a disc degeneration rate λ_1_. Once in compartment *D*_0_, a return to compartment *N* is not permitted (i.e., disc degeneration is uni-directional and irreversible).
5. *D*_0_ → *D*_1_: Asymptomatic individuals who reside in compartment *D*_0_ can move on to compartment *D*_1_ with a symptom-attack rate λ_2_.
6. *D*_1_ → *D*_0_: Symptomatic patients in compartment *D*_1_ can recover with a symptom-resolution rate λ_3_ and return back to compartment *D*_0_. The bi-directional flows between the last two compartments (*D*_0_ and *D*_1_) account for the recurrent nature of low back pain. Depending on the goal of a particular clinical or research application, *D*_0_ can be approximately composed of those individuals who are presently asymptomatic, but have a prior back pain history.

### Paired prevalence predictions

Quantitative relationship among the three compartment variables, *x* (*t*), *y* (*t*), and *z* (*t*), are described by a system of differential equations (see Appendix A). Their solutions are exponential functions of age *t*. Continuously and concurrently observing their age-dependent trajectories in a stable population would be ideal; however, conducting such a large and prolonged cohort study is expensive and challenging. Fortunately, from these population-based variables, we can derive a pair of prevalence functions that are easier to observe and verify (Appendix B):

$$ {z}^{*}(t)\cong \alpha +\beta \left(1 - {e}^{-{\lambda}_1\left(t-{t}_0\right)}\right), $$

$$ {y}^{*}(t)\cong \frac{\left(1-\beta \right)\left(1 - {e}^{-{\lambda}_1\left(t-{t}_0\right)}\right)}{\ {e}^{-{\lambda}_1\left(t-{t}_0\right)}+\left(1-\beta \right)\left(1 - {e}^{-{\lambda}_1\left(t-{t}_0\right)}\right)}. $$

Here z^*^(*t*) is the population prevalence of low back pain at age *t*; α is the age-independent component; $\beta =\frac{\lambda_2}{\lambda_2+{\lambda}_3}$ is the symptom expression fraction among individuals who have disc degeneration; λ_1_ is the disc degeneration rate that solely determines the age dependence of the low back pain prevalence; and *t*_0_ is the transition age at which intervertebral discs lose the growth potential and begin degenerating.

The companion function y^*^(*t*) is the percentage of disc degeneration detected through a lumbar MRI among asymptomatic individuals of age *t*. In general, observing y^*^(*t*) requires a lumbar MRI and the loss of T2-based signal intensity (Pfirrmann et al. 2001), which is considered to be a more sensitive indicator for disc degeneration than other late-stage signs such as disc herniation or Modic-type change (Modic et al. 1988). Although the algebraic expression of y^*^(*t*) appears slightly more complicated and is affected by λ_1_ and (1-β), both y^*^(*t*) and z^*^(*t*) are derived from the same population-dynamics model. Collectively they illustrate the degeneration-dependent and highly recurrent nature of low back pain.

### The duration factor μ

In many clinical studies, a low back pain patient is considered an acceptable case only if she (or he) has had “persistent back pain for μ days”. Although introducing such a duration factor μ into the process of case (or control) ascertainment has certain benefits, its presence complicates the comparative analysis of independently conducted prevalence studies. This is because the use of a longer duration μ in a case definition means fewer people would meet the residence criteria to stay in compartment *D*_1_. So more individuals will be assigned from compartment *D*_1_ to compartment *D*_0_. On the other hand, when the duration factor μ is used to more selectively define the asymptomatic individuals (e.g., non-cases must be “pain-free for μ days”), fewer individuals will be eligible for residence in compartment *D*_0_.

The seemingly artificial influence of the duration factor μ on the back pain prevalence can be arithmetically adjusted for. Specifically, if we know the value of the duration factor μ and the transition rate λ_3_ (or λ_2_), we can convert the symptom expression fraction β into a duration-adjusted β^*^. For instance, if the use of a duration factor μ is λ_3_-centric (i.e., a case definition is based on having “persistent back pain for μ days”; or logically equivalent, a non-case is defined as having “any pain break during μ days”), the duration-adjusted symptom expression fraction β^*^ is approximately equal to $\beta\ {e}^{-{\lambda}_3\mu }$. Similarly, if the use of a duration factor μ is λ_2_ -centric (i.e., “pain-free for μ days” is used to define the non-cases; or logically equivalent, “any back pain during μ days” for the cases), we have ${\beta}^{*} \cong \kern0.37em \beta + \left(1-\beta \right)\left(1-{e}^{{}_{{}^{-\lambda }{2}^{\mu }}}\right)$. Noteworthy among the published prevalence studies that have adopted a λ_2_ -centric duration factor is the result that the 1-month period prevalence is about twice the point prevalence (Hoy et al. 2012; Thiese et al. 2014). Assuming α is negligible, this implies β^*^/β ≅ 2.

### Parameter estimation

The method of least squares is used to minimize the error function ${\displaystyle {\sum}_t}{\left(O(t)-E(t)\right)}^2$. Here *O* (*t*) is the number of observed cases in the age- *t* group; *E* (*t*) (=*P* (*t*) × z^*^(*t*)) is the number of expected cases in the same age group (*P* (*t*) is the total number of individuals); ∑_*t*_ represents summation over the entire age range. Numerical implementation is completed in MS Excel spreadsheet. The known percentage of cases who have no disc degeneration (about 7 %; see Hancock et al. 2012) is also utilized to estimate the age-independent component α in the prevalence function z^*^(*t*) because α and *t*_0_ cannot be separately calculated from the prevalence data alone.

## Results

### Analysis of prevalence data

**Fig. 2:**
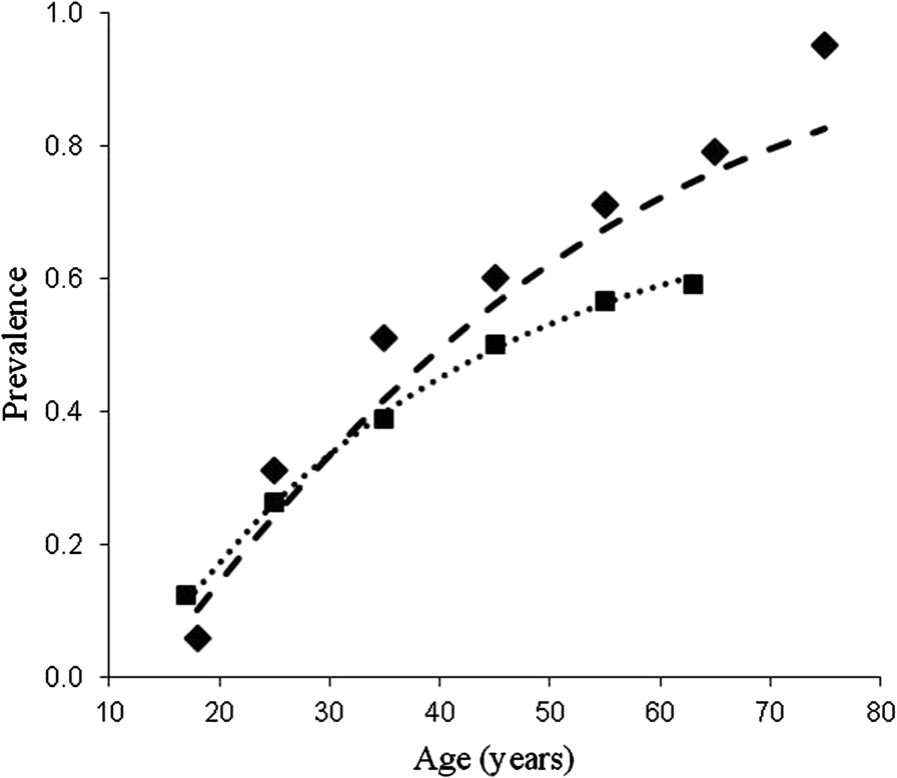
Changing prevalence of low back pain (filled squares and dotted line) and asymptomatic disc degeneration (filled diamonds and dashed line) with age. The filled squares represent the observed prevalence of low back pain among the Hungarian population (Horváth et al. 2010). The dotted line represents the result of curve fitting with the prevalence function ${z}^{*}(t)=\alpha +\beta \left(1 - {e}^{-{\lambda}_1\left(t-{t}_0\right)}\right)$ (α = 0.031, β = 0.698, λ_1_ = 0.0344, *t*_0_ = 13.3; *R* ^2^ = 0.998). Filled diamonds are based on the graph (percentage of disc degeneration among asymptomatic women) of Powell et al. (1986). The prediction (dashed line) is based on the function ${y}^{*}(t)=\frac{\left(1-\beta \right)\left(1 - {e}^{-{\lambda}_1\left(t-{t}_0\right)}\right)}{\ {e}^{-{\lambda}_1\left(t-{t}_0\right)}+\left(1-\beta \right)\left(1 - {e}^{-{\lambda}_1\left(t-{t}_0\right)}\right)}$ (*R* ^2^ = 0.978). Note that the parameter values of *y* ^*^(*t*) are taken from *z* ^*^(*t*). The only adjustment made is to the symptom expression fraction β (normalized for a predefined duration factor μ; see main text for more details)

The age-specific prevalence data we have chosen to analyze is published recently by Horváth et al. (2010). This is one of the largest surveys on low back pain prevalence, involving a random sample of 10,000 people (aged 14–65) from Hungary. The data set was divided into six age intervals and the percentage of people who reported having low back pain in the last month at the time of the questionnaire-based survey was tabulated for each of them. Curving fitting with the prevalence function z^*^(*t*) (Fig. 2; *R*^2^ = 0.998) yields the following numerical result:

$$ {z}^{*}(t)=0.031+0.698\left(1 - {e}^{-0.0344\left(t-13.3\right)}\right). $$

Here α = 0.031, β^*^ = 0.698, λ_1_ = 0.0344/year (1/λ_1_ = 29.1 years is the residence time), and *t*_0_ = 13.3 years.

### Analysis of MRI data

To verify y^*^(*t*), we have examined the data from Powell et al. (1986) who reported on the percentage of disc degeneration among 302 asymptomatic women (age 16–80) detected with lumbar MRI. Their original data points were given in a graph format which is re-digitized to capture the numerical values. Both Powell et al. (1986) and Horváth et al. (2010) had used a λ_2_ -centric duration factor in their sampling procedures. So a numerical transformation of the symptom expression fractions can be used to make the two studies directly comparable. In Horváth et al., the duration factor was μ = 1 month (i.e., an acceptable case was defined as having “pain in the last month”). In Powell et al., the duration factor was μ ≅ 0 (i.e., an accepted non-case was defined as having “no symptoms relating to spinal disease at the time of examination”). Given that the estimated symptom expression fraction β^*^ for Horváth et al. is 0.698, the adjusted symptom expression fraction for the Powell et al.’s study becomes β ≅ β^*^/2 = 0.698/2 = 0.349. The percentage of disc degeneration among the asymptomatic women is then

$$ {y}^{*}(t)=\frac{0.651\left(1-{e}^{-0.0344\left(t-13.3\right)}\right)}{e^{-0.0344\left(t-13.3\right)}+{0.651}^{\left(1-{e}^{-0.0344\left(t-13.3\right)}\right)}.} $$

Contrasting this projection with the observed data from Powell et al. (1986), we notice that the projected percentage of disc degeneration shifts slightly downward, but its age-related trend agrees quite well with the observed aging pattern (see Fig. 2; *R*^2^ = 0.978).

## Discussion

Disc degeneration is irreversible. Although the decade-long time scale and the association with low back pain being expressed only through reversible and recurrent pain episodes make it difficult to evaluate its etiological contribution, the decay process itself is in theory amenable to conventional survival analysis. Several research groups have indeed used lumbar MRIs to identify individuals free of disc degeneration and monitor them over time for disc-signal change. In one of the earliest longitudinal studies of this kind, Salminen et al. (1995) uncovered 5 new cases out of 43 at-risk adolescents during a 3-year follow-up. In another study, Elfering et al. (2002) detected 4 new cases among 21 at-risk adults at a 5-year recheck. In a more recent study, Carragee et al. (2006) reported that 10 % of the at-risk subjects had developed new disc degeneration over a 5-year period. Pooling these observations together, we are able to calculate an average degeneration rate λ_1_ ≅ 0.0348/year. This longitudinally measured value is essentially the same as that (0.0344/year) derived from the modeling analysis of the back-pain prevalence data. The consistency among the three different types of studies (i.e., cross-section survey, lumbar MRI of asymptomatic controls, and MRI follow-up of normal individuals) signifies the notation that disc degeneration underlies the age dependence in low back pain.

Although only the relative ratio β. is present in the prevalence functions z^*^(*t*) and y^*^(*t*), knowing the individual values of λ_2_ and λ_3_ helps determine when the approximations (λ_2_ > > λ_1_ and λ_3_ > > λ_1_; see Appendix A) remain valid. To accomplish this goal, two different approaches can be considered. The first one is to compare the 1-month period prevalence with the point prevalence. For instance, from ${\beta}^{*} \cong \kern0.37em \beta +\left(1-\beta \right)\left(1 - {e}^{-{\lambda}_2\mu}\right)=0.698$ and *β* ≅ β^*^/2 = 0.349, we can roughly infer λ_2_ = 9.33/year (the residence time = 0.11 year) and λ_3_ = 17.4/year (the residence time = 0.057 year). Alternatively, clinical studies that prospectively monitor the time to pain resolution (or the time to pain recurrence) can be used to calculate the symptom attack rate λ_2_ and the symptom resolution rate λ_3_. For instance, assuming that 80 % of low back pain patients recover within 6 weeks of the symptom onset (Waddell 1987) and that the recovery time follows an exponential distribution, we have λ_3_ = 13.9/year (the residence time = 0.072 year). Since it is also known that β = 0.349, we can further infer λ_2_ = 7.5/year (the residence time = 0.13 year). The noted difference in these estimates of λ_2_ and λ_3_ is not surprising given that non-physiological factors like the pain rating scale or cultural norms affect the classification of a pain status. Nevertheless, the resolution-then-recurrence dynamics of low back pain have unambiguously a time scale (i.e., weeks or months) that is 2–3 orders of magnitude shorter than that of disc degeneration (i.e., decades).

The symptom expression fraction β (=λ_2_/λ_2_ + λ_3_) is mechanistically distinct and algebraically separable from the disc degeneration rate λ_1_. It alone determines the prevalence (recurrence risk) of low back pain among a group of individuals who have disc degeneration. Because individuals who reside in compartments *D*_0_ and *D*_1_ are dynamically exchangeable at very fast rates (i.e., λ_2_ > > λ_1_ and λ_3_ > > λ_1_), being provisionally present in one compartment does not preclude an individual from returning to the other compartment at a later time. Therefore, even though the relative size of the two compartments is stable, the equilibrium is maintained by frequent inter-compartment exchanges. In the special case β = 1, *D*_1_ merges into *D*_0_ and the three-compartment model is reduced to a conventional survival model.

The influence of a duration factor on the prevalence of low back pain is widely known. Our modeling analyses indicate that existing usages of the duration factor μ can be classified into two discernible categories (λ_2_-centric versus λ_3_-centric). Within each category, a conversion formula can be used to normalize β and facilitate quantitative between-study comparison. In an active effort to standardize the outcome measures for low back pain research, Deyo et al. (1998) recommended μ = 7 days. The way the survey question was worded (“during the past week, how bothersome have the following symptoms been? …”) indicates that it is a λ_2_-centric definition. Whether or not a separate λ_3_-centric definition should be established is an open question for future research.

The estimated transition age (*t*_0_ = 13.3 years) at which the lumbar intervertebral discs stop the growth process and enter into the disc degeneration phase is a fundamental developmental-biology parameter. To our limited knowledge, there are few comparative studies available to corroborate this value. Salo et al. (1995) reported a study of 32 patients and 49 controls under 15 years old with lumbar MRI scans and they noted that disc degeneration is seldom found in patients under 10 years old. Although their observation does not contradict our prevalence-based estimation, more studies are clearly needed in this area.

A number of cohort studies have attempted to quantify the association between disc degeneration and low back pain using either an odds ratio or a relative prevalence ratio (reviewed by Steffens et al. 2014). However, their findings are too heterogeneous to be conclusive. Using the population-dynamics model as a conceptual guide, we can observe how the co-existence of two time scales contributes to this heterogeneity in a hypothetical cohort study with two groups of study subjects, one with disc abnormalities on a baseline lumbar MRI (group-*D*_0_) and the other without (group-*N*). After a period ∆*t* of several years, we recheck both groups to measure the back pain prevalence (*β* for group-*D*_0_; and $\alpha +\beta \left(1 - {e}^{-{\lambda}_1\varDelta t}\right)$ for group-*N*). The relative prevalence ratio is computable as $\frac{1}{\left(\alpha /\beta \right)+\left(1 - {e}^{-{\lambda}_1\varDelta t}\right)}$. Here the parameter (α/β) primarily reflects the effect of pain recurrence and is highly influenced by the duration factor μ. For a constant λ_1_ (scaling to decades), a longer duration ∆*t* of follow up decreases the prevalence ratio. Additionally, by recruiting research subjects from adult populations, many cohort studies inflate λ_1_ by assigning individuals with milder disc abnormalities into group-*N* rather than group-*D*_0_. This misclassification also decreases the prevalence ratio. Explicitly recognizing the significance of time scale difference should help improve the design of future cohort studies.

To construct a dynamic model that is simple and yet still capable of explaining the essential features of low back pain epidemiology, we have chosen to ignore the progressive nature of disc degeneration and also similarly lump together all back pain complaints (with or without nerve radiculopathy) as one clinical entity. This provision allows us to focus on the most critical connection between disc degeneration and low back pain, and yet minimize various non-essential mechanistic pathways among the model’s building blocks. The resultant model is surprisingly robust in the sense that adding a non-zero rate of transition directly from compartment *N* to compartment *D*_1_ would not alter any of the predictions made. However, for more complex applications, further relaxing some of the model’s assumptions is likely necessary. For example, the number of compartments may need to be extended to accommodate the scenarios that disc degeneration can continuously advance into other abnormalities, producing chronic pain or sciatica, etc. Also, many clinical studies have a small sample size and a probabilistic approach (with flexible event-time distributions) may be necessary. To evaluate the effect of genetic predispositions, treatment regimens and physical activities, the transition rates may become subject-dependent and time-dependent.

## Conclusion

In summary, the complexity of low back pain epidemiology results from the co-existence of two different time scales: the slow dynamics of disc degeneration and the fast dynamics of pain recurrence. Each time scale is best measured within a specific age range or a specific follow-up period. Disc degeneration is well under way and most discernible among the young and continuously rises among the older adult population. It lays the foundation upon which the fast dynamics of pain recurrence emerge in succession. As such, it should be recognized as the predominant cause of low back pain.

## Appendix A

*x* (*t*), *y* (*t*), and *z* (*t*) obey a system of linear differential equations:

$$ \frac{dx(t)}{dt}=-{\lambda}_1x(t), $$

$$ \frac{dy(t)}{dt}={\lambda}_1x(t)-{\lambda}_2y(t)+{\lambda}_3z(t), $$

$$ \frac{dz(t)}{dt}={\lambda}_2y(t)-{\lambda}_3z(t). $$

Assume that all the coefficients are constant and that the initial conditions *x* (*t*_0_) = *x*_0_, *y* (*t*_0_) = 0, and *z* (*t*_0_) = 0, one can easily obtain the following solutions using a number of standard techniques:

$$ x(t)={x}_0\ {e}^{-{\lambda}_1\left(t-{t}_0\right)}, $$

$$ y(t)={x}_0\frac{\lambda_3\left(-{\lambda}_1+{\lambda}_2+{\lambda}_3\right)\left(1-{e}^{-{\lambda}_1\left(t-{t}_0\right)}\right)-{\lambda}_1{\lambda}_2\left({e}^{-\left({\lambda}_2+{\lambda}_3\right)\left(t-{t}_0\right)}-{e}^{-{\lambda}_1\left(t-{t}_0\right)}\right)}{\left(-{\lambda}_1+{\lambda}_2+{\lambda}_3\right)\left({\lambda}_2+{\lambda}_3\right)}, $$

$$ z(t)={x}_0\frac{\lambda_2\left({\lambda}_2+{\lambda}_3\right)\left(1-{e}^{-{\lambda}_1\left(t-{t}_0\right)}\right)-{\lambda}_1{\lambda}_2\left(-{e}^{-\left({\lambda}_2+{\lambda}_3\right)\left(t-{t}_0\right)}+1\right)}{\left(-{\lambda}_1+{\lambda}_2+{\lambda}_3\right)\left({\lambda}_2+{\lambda}_3\right)}. $$

Recognizing that λ_2_, λ_3_ >> λ_1_ (i.e., the symptom-attack rate and the symptom-resolution rate are generally much larger than the disc degenerate rate), we can also approximate *y* (*t*) and *z* (*t*) with the following ($\beta =\frac{\lambda_2}{\lambda_2+{\lambda}_3}; - {\lambda}_1+{\lambda}_2+{\lambda}_3\cong {\lambda}_2+{\lambda}_3;\ \frac{-{\lambda}_1{\lambda}_2}{\left(-{\lambda}_1+{\lambda}_2+{\lambda}_3\right)\left({\lambda}_2+{\lambda}_3\right)}\cong 0$):

$$ y(t)\cong {x}_0\left(1-\beta \right)\left(1 - {e}^{-{\lambda}_1\left(t-{t}_0\right)}\right), $$

$$ z(t)\cong {x}_0\beta \left(1 - {e}^{-{\lambda}_1\left(t-{t}_0\right)}\right). $$

## Appendix B

The population-dynamics model solved in Appendix A can be modified to produce a number of related predictions that are more applicable in clinical studies.

### Cross-section study

The typically observed variable is the age-specific prevalence of low back pain

$$ {z}^{*}(t)\cong \alpha +\frac{z(t)}{x_0}=\alpha +\beta \left(1 - {e}^{-{\lambda}_1\left(t-{t}_0\right)}\right). $$

Here a small constant term α is included to account for the age-independent origin of low back pain; β is the symptom expression fraction among individuals with disc degeneration (β >> α); λ_1_ is the disc degeneration rate; *t*_0_ is the transition age at which the lumbar intervertebral discs stop the growth process and begin degenerating.

### Case–control study

When lumbar MRI is used to evaluate disc degeneration, the already elevated percentage of disc degeneration among the cases may be found to increase only slightly with age *t*, $\frac{\beta \left(1 - {e}^{-{\lambda}_1\left(t-{t}_0\right)}\right)}{\alpha +\beta \left(1 - {e}^{-{\lambda}_1\left(t-{t}_0\right)}\right)}$. Moreover, the most dramatic increase with age should be observed among the asymptomatic controls:

$$ {y}^{*}(t)=\frac{y(t)}{x(t)+y(t)}=\frac{\left(1-\beta \right)\left(1 - {e}^{-{\lambda}_1\left(t-{t}_0\right)}\right)}{\ {e}^{-{\lambda}_1\left(t-{t}_0\right)}+\left(1-\beta \right)\left(1 - {e}^{-{\lambda}_1\left(t-{t}_0\right)}\right)}. $$
